# Assessing the impacts of conservation volunteering on participant wellbeing: a systematic review protocol

**DOI:** 10.12688/f1000research.113630.2

**Published:** 2023-03-06

**Authors:** Hanna Nuuttila

**Affiliations:** 1 College of Science, Swansea University, Swansea, Wales, SA2 8PP, UK

**Keywords:** conservation, volunteering, community, wellbeing, citizen science

## Abstract

**Background:** Volunteers and citizen scientists have become an essential element of most nature conservation and restoration activities due to lack of resources but also due to the wish to engage and interact with local communities where conservation activities take place. Environmental or conservation volunteering is also considered to be a key resource in achieving much needed, ambitious nature restoration programs. Practical conservation work and various forms of environmental enhancement along with recreational and therapeutic use of natural or green and blue spaces have been studied for some time. The value of volunteers and the work is widely acknowledged but few studies have been carried out on the impacts of participating on the volunteers themselves. Using this protocol, a study will be undertaken to assess how impacts of participation have been assessed and reported in the literature; what these reported impacts are; how these are related to reported barriers and motivations for volunteering and whether they are affected by the region or country of study.

**Methods:** This paper will identify studies that have described and assessed impacts of conservation and restoration volunteering on participants at an individual level, with a specific focus on physical, mental or societal wellbeing of individuals. Representative studies were sought from major search engines and relevant stakeholder publications, including both peer-reviewed and ‘grey literature’ in predominantly English language publications, published between 2000 and 2020. A priori inclusion criteria consisted of those publications and reports on studies with volunteer and community participants and which described impacts of, motivations for and barriers to participation. After a critical appraisal, a total of 105 articles were selected for further analysis to provide a narrative and mixed methods synthesis of the evidence base.

## Introduction

### Background/rationale

We intuitively know that healthy ecosystems are essential for human wellbeing. ^1^^,^^2^ This has become widely accepted and reflected both in international policy initiatives as well as in the vision of major international and national conservation organisations. ^3^ The effect of environmental enhancement along with recreational and therapeutic use of natural or green and blue spaces have been studied for some time. ^4^^–^^6^ Nature-based interventions, exposure to natural environments and ‘green exercise’ have shown to be beneficial to health and wellbeing regardless of age, gender, ethnicity or social status. ^7^ The natural environment is accepted as a vital provider of health and other environmental services whilst life-style related conditions and illnesses influenced by a lack of physical activity, links to natural places, and links to community and people amount to around £180 billion a year in the UK alone. ^8^ The theoretical framework behind this project is based on the Green Mind Theory put forward by Pretty *et al*., 2017, ^9^ which links the human mind with the brain and body and connects the body with natural and social environments with reciprocal processes: environments shape bodies, brains, and minds; minds change behaviours that shape social interactions and natural capital.

Volunteering and volunteers have become an essential element of most nature conservation and restoration activities due to a lack of resources ^10^^–^^12^ but also due to the requirement to engage and interact with local communities where conservation activities take place. Volunteering is acknowledged by UK national governments as a valuable resource to achieve nature conservation and regeneration targets ( www.nationalnatureservice.org). The value of volunteers and the work is widely acknowledged but few studies have been carried out on the impacts of participating on the volunteers themselves. ^13^ While many studies report the use of volunteers in conservation projects and describe the value of volunteers to conservation efforts, they rarely describe the impacts of volunteering to volunteers themselves or to the wider community. Qualitative evidence shows that environmental enhancement and conservation activities are valued by participants and contribute to their health and wellbeing, but quantitative evidence is in most part lacking. ^5^^,^^6^ As the quality of the evidence is perceived to be low, most reviews as well as individual studies have not been able to draw definite conclusions. Furthermore, few studies have assessed these effects systematically or even described the ways in which these impacts have been assessed.

This study will review the most recent research on the topic to better understand the impact of conservation activities on participating volunteers, how these impacts of participation have been assessed and reported in up-to-date literature; what these reported impacts are; how these are related to reported barriers and motivations for volunteering and whether they are influenced by the region or country of study. The study will also critically assess the methods used in selected studies to describe and examine the impacts and make recommendations for future studies in the field. As volunteers will continue to be required to support critical nature restoration projects, it is vital to understand how volunteering may impact participants. Furthermore, understanding the positive co-benefits of nature related activities such as conservation volunteering can be used to create pathways for multi-solving interdisciplinary eco-societal issues and fast-tracking these into action.

Various stakeholders, both academic and non-academic were approached for assistance, including members of the UK health and wellbeing organisation (NHS), volunteer sector, conservation, and community development. Several people were able to assist and contribute to the theoretical framework, question formulation and the literature search strategy, ensuring a thorough understanding of the key concepts relevant to the review. ^14^

One of the most difficult to define concepts was ‘human wellbeing’. As our understanding of wellbeing has evolved in psychology, sociology and economics, so has our way of defining and assessing it in conservation practice and policy. Understanding human wellbeing is now a critical component in sustainably managing the environment. Biedenweg et al. identify six human wellbeing indicators (psychological, physical, social, cultural, economic, governance) affected by the environment and useful in studies policy and management. ^15^ It is acknowledged that human wellbeing has both objective and subjective aspects. ^16^ The objective components (material and social attributes) are easily measurable such as our jobs and incomes, homes, places we live in, our environment, available services, infrastructure so on. Equally important is the subjective, perceived wellbeing which can only be described by the individual. These include job or health satisfaction, our feelings of belonging, empowerment, available choices as well as our perceived level of mental wellbeing, our relationships and confidence in society and civic institutions around us. At its most simple form, human wellbeing in conservation has been defined through three conditions: meeting needs, pursuing goals and experiencing a satisfactory quality of life. ^17^

Crucially, wellbeing is now acknowledged to being not just individually constructed and perceived ^16^ but also a social and cultural construct, with differing aspects across spatial and temporal scales, even if some components are universally accepted. ^18^^–^^21^

Here we used a multidimensional framework for wellbeing, incorporating all aspects of our lives including social and cultural, environmental and physical, economic and material, and psychological including emotional and spiritual wellbeing.

### Objectives of the review

The main objective of this review was to understand whether participation in voluntary conservation and restoration programs has specific impacts on the participants and their communities. The primary research question was:

**What are the impacts (O) (positive or negative) of voluntary participation (I) in conservation and restoration activities to the participating volunteers (P)?**

**Figure 1.**
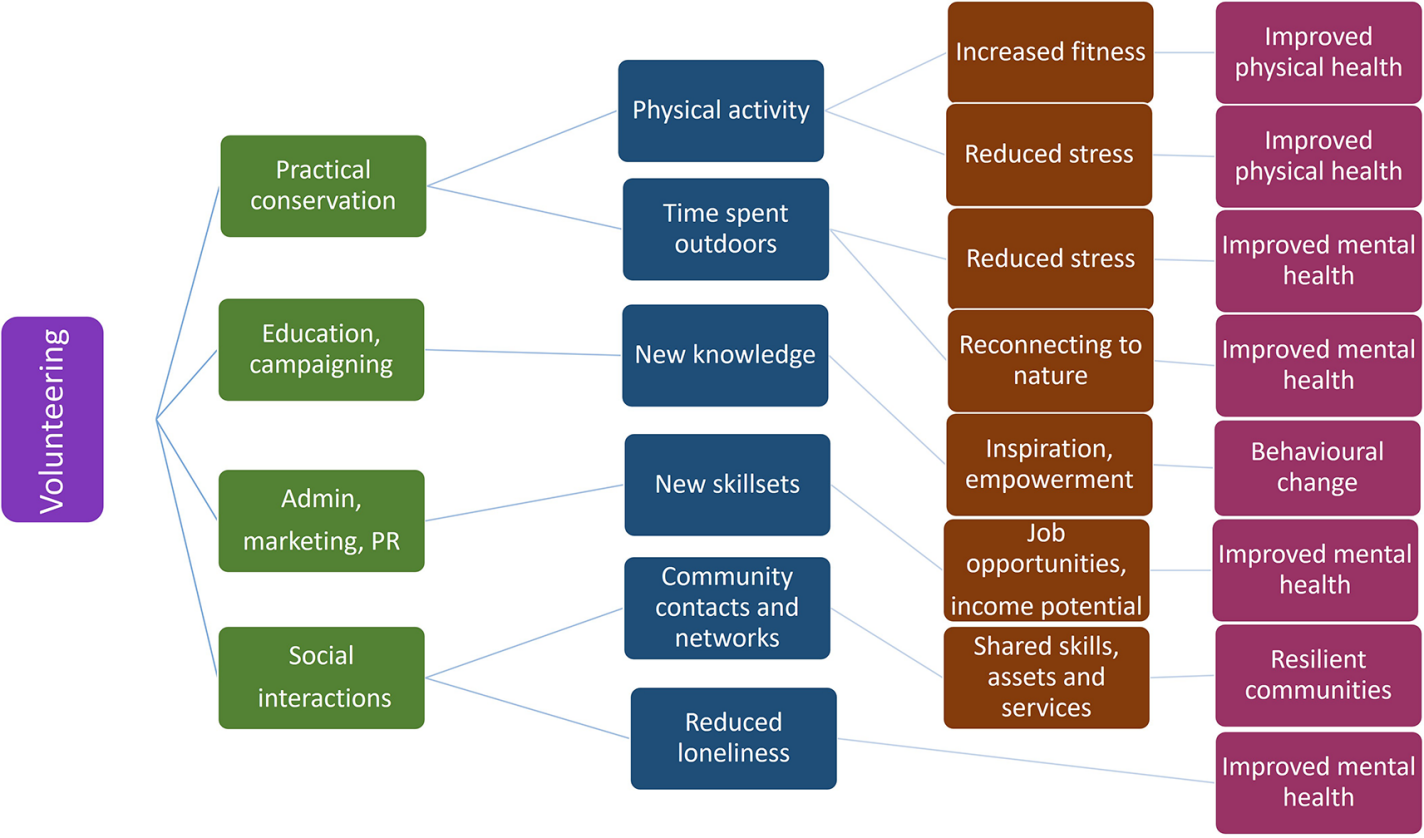
Conceptual flowchart illustrating potential positive impact pathways from volunteering to the participating individual based on theory of change process.

In addition to the primary research question other related questions were identified. These included questions on how the impacts of participation had been defined and assessed; what methods have been used to do so and what variables may affect the extent of these impacts or how they have been evaluated and reported. Conceptual framework for potential impact pathways is depicted in Figure 1.

The secondary research questions were:

• How many studies were identified that specifically describe impacts on participants and/or volunteers?
• How many of those impacts specifically describe or refer to the concept of ‘wellbeing’?
• If ‘wellbeing’ is discussed, described, or assessed, how has it been defined? What aspects of wellbeing have been included?
• How have impacts on participants and/or volunteers been collected, measured, or evaluated?
• Has volunteering or participating been shown to have an impact on ‘wellbeing’ or any of its aspects?
• Has participation in conservation/restoration projects been shown to improve any aspect of individual wellbeing?
• Has participation in conservation/restoration projects been shown to achieve improved wellbeing for communities?
• What motivates people to volunteer?
• What barriers have been described which might prevent people from volunteering?
• What challenges may arise within volunteering situation that may prevent people from continuing their volunteering commitment?
• Where in the world have studies included in this review been conducted? Where are the institutions situated to which the first authors of included studies have been affiliated with? Does this influence types of impacts, barriers or motivations described?

Definitions of the question components:

The **subject/population (P)**, or the unit of study was any identified habitat or species conservation or restoration project with a definite aspect of community and/or volunteer participation described in the literature.

The **intervention (I)** was the volunteer participation, and the outcome (O) was the effects on participants’ or perceived mental, physical, social and economic wellbeing, both at individual as well as community level.

The review’s focus was specifically the described (subjective) perceptions of individuals who have participated in volunteering activities. As such, it was not possible to find a suitable **comparator**, such as review of volunteering impacts from non-conservation projects, that would have provided adequate, reliable data for comparison.

## Protocol

The review was conducted following the methodological guidelines set by the Centre for Evidence-based Conservation (CEC) at Bangor, Wales, UK ^22^^–^^24^ and the PRISMA-P guidelines. ^25^^,^^26^ The study arose from a restricted situation during COVID regulations and is not a standard systematic review in a sense of having resources for a large team of researchers. The study was conducted mostly by one researcher but aiming for the higher standards than that of a typical literature review.

### Searches

Six bibliographic databases were used for this review: ISI Web of Science, JSTOR, Scobus, ScienceDirect, Google Scholar and Lens. The search was conducted for years between 2000 and 2020 as 20 years’ worth of data was the deemed the maximum period to limit excessive data from earlier years when a pilot search had shown that ‘wellbeing’ was very rarely linked with volunteer research.

The search strings used are listed below:

*“conservation” OR “community conservation” OR “community based conservation” OR “community-based conservation” OR “volunteer conservation” OR “participatory conservation” OR “citizen science” AND “volunteer” OR “volunteering” OR “wellbeing” OR “well-being”*

In addition, limited searches were conducted for “impacts of conservation”, “impacts of conservation volunteering”, “impacts of community-based conservation”, “co-benefits of conservation”, “co-benefits of conservation volunteering”, “conservation volunteering” AND “happiness”, “conservation volunteering” AND “health” to check how well the general search string were capturing the intended literature.

English language was used in bibliographic database searches as well as organizational website searches and web-based search engines. Organisational websites were searched for additional literature, see Table 1. The number of databases used, and the grey literature searched, hopefully ensures the comprehensiveness of the search strategy. Due to lack of resources, there are currently no plans to update the searches during the conduct of the review.

**Table 1.** Organisational websites consulted for additional literature.

#	Organisation	Website
1	National Resources Wales	https://naturalresources.wales
2	The Wildlife Trust	https://www.wildlifetrusts.org/
3	British Trust for Conservation Volunteers	https://www.tcv.org.uk/
4	Marine Conservation Society	https://www.mcsuk.org/
5	Department of Conservation, New Zealand	https://www.doc.govt.nz/
6	Scottish Forestry Trust	https://www.scottishforestrytrust.org.uk/

Inherent meta-bias is present through the use of mostly English language literature. Although attempts are made in the analysis to identify countries where studies are conducted as well as the nationalities of first authors, it is not possible to disentangle the language bias from the dataset. This will be discussed in the final results.

**Figure 2.**
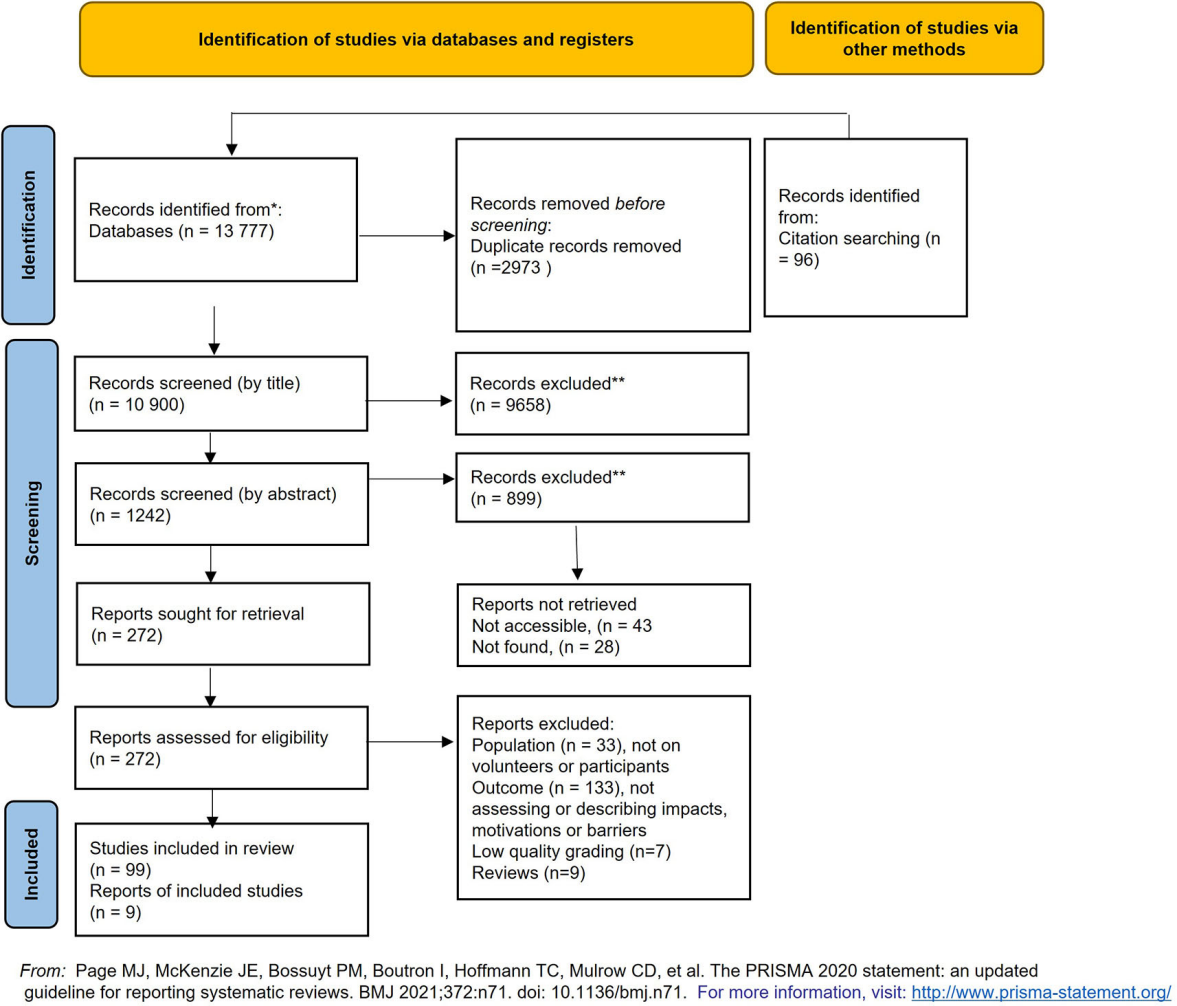
Prisma-P diagram for the literature search and appraisal process.

A total of 13,777 documents were identified from web-based database searches, this figure was reduced to 10,804 after duplicates were removed using the ‘duplicate’ search in Excel tables. Finally, the list was organised in an alphabetical order and visually checked for duplicates. An additional 96 articles were sourced from organisational websites, key-literature references and publication depositories and added to the search data ( Figure 2).

### Article screening and study inclusion criteria

A priori methodology for screening the articles was conducted in three stages. The initial screening covered only the title of each document, article or a report. Irrelevant titles, as well as all further duplicates were deleted.

A total of 1242 articles and documents remained after the initial title screening ( Figure 2). Articles were kept for further reading if deemed otherwise interesting and relevant to the project.

Title screening consisted of checking the title against the following criteria and whether it alluded that the paper would discuss any of the following: conservation/restoration/rewilding project/natural resource management/volunteer participants/community conservation/community participation/citizen science/impacts or wellbeing linked to conservation, environment and volunteering in any form/challenges, barriers, motivations, attitudes, or incentives to volunteering or community based conservation or participatory experience.

If it wasn’t not clear whether or not the article might discuss the above, it was kept for further screening. In addition, included for further reading, were titles that alluded to the above topics which could be discussed in the text, specifically when these were titled as reviews or analyses.

The articles and studies identified by their title were downloaded where possible and if not found or the weblinks were unavailable the authors were contacted, and copies requested.

The checklist against which each article was compared included the following:

• Does the article discuss a conservation/restoration/rewilding project/natural resource management? (YES/NO)
• Does it also describe, discuss or assess volunteer participants/community conservation/community participation/citizen science? (YES/NO)
• And/or does it also describe, discuss or assess impacts or wellbeing linked to conservation, environment and volunteering in any form? (YES/NO)
• Furthermore, does it discuss challenges, barriers, motivations, attitudes, or incentives to such project – anything that would indicate a discussion of the participatory experience, not just merely that volunteers were used to collect data? (YES/NO)

In addition, included for further reading, were titles that alluded to the above topics which could be discussed in the text, specifically when these were titled as reviews or analyses. Abstracts that related exclusively to ecotourism or tourism were not included.

A total of 272 articles went through an additional abstract screening stage. After which 106 articles remained for more detailed assessment ( Figure 2).

### Consistency checking

A Cohen’s kappa analysis was conducted on a selection of (n=115, 10%) titles between the first reviewer and an additional reviewer to check for consistency of the filtering approach.

Formula used was as follows

K=(Pr(a)-(Pr(e)) / (1-Pr(e)). The resulting Kappa coefficient was 0.7, indicating a moderate to good agreement between the title selection of the reviewer.

### Critical appraisal strategy

The critical appraisal strategy consisted of assessing the quality or the ‘internal validity’ of each study based on the extent, repeatability and clarity of methodology description (“well described”, “limited description”, “not described”).

The generalisability of the ‘external validity’ was ascertained based on the sample size of each study, where studies with less than 20 samples, combined with a limited description of methodology were classified as ‘low’ quality (n=2). Studies with 20 or more samples but limited methodological account (n=30) were examined case by case. Initially six of these studies were subjectively given a ‘high’ quality classification but this was downgraded to ‘low’ after further reflection as no objective, repeatable threshold could be described.

Studies with no description of research methodology were excluded from the outset. Studies were further excluded from the analysis if a low sample size was combined with a methodology that was not adequately described resulting in ‘low’ quality data with lack of methodological description ( Table 2). Not all ‘low’ quality reports were excluded. Of the final 88 studies, 51 were considered ‘high’ quality and 37 ‘low’ quality.

**Table 2.** Research quality matrix and scoring used to assess the validity of each study at critical appraisal stage.

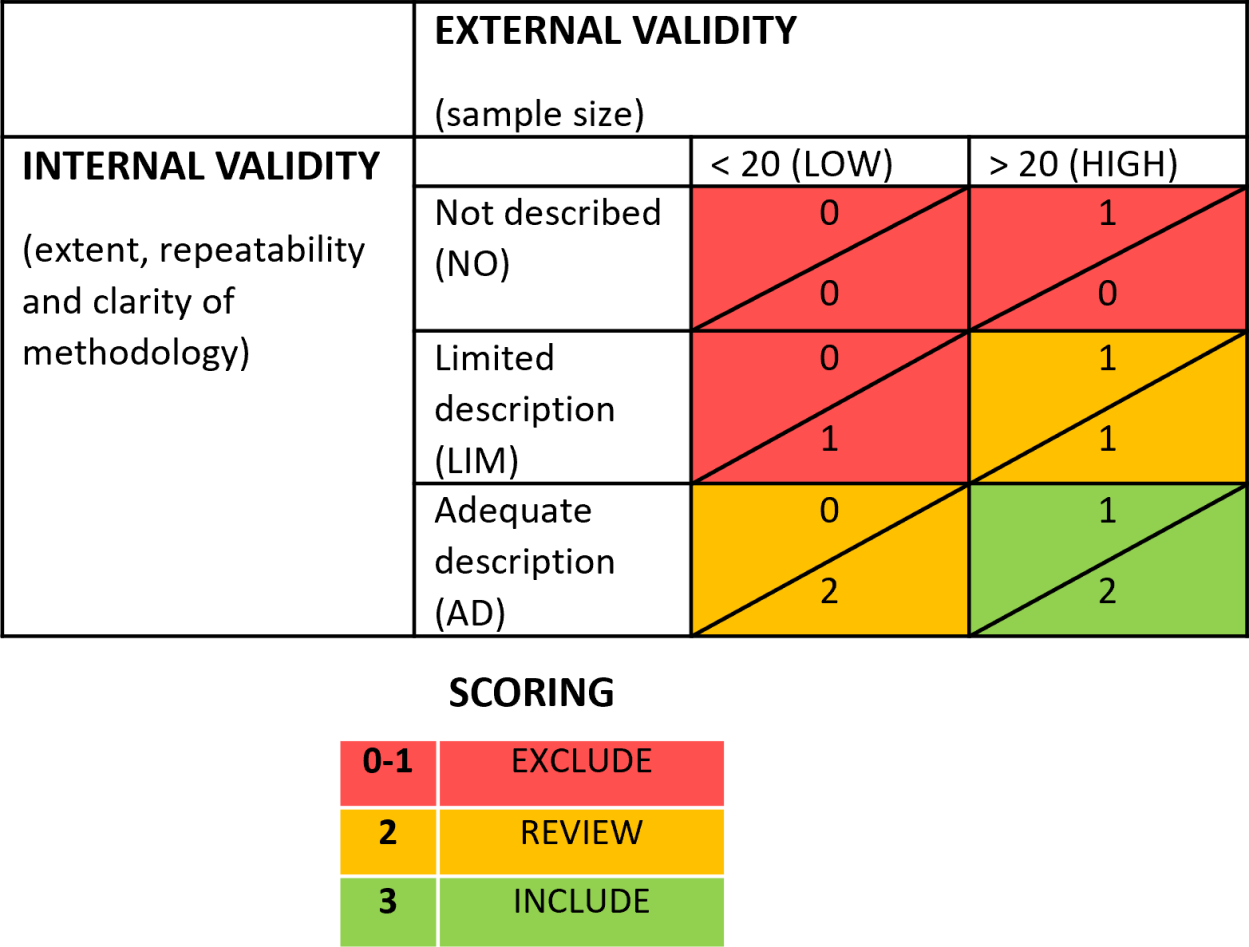

The sample size will be used as a descriptor for the resulting synthesis and processed further when the validity of the evidence base is weighed up and discussed. Reviews were collated for a separate narrative synthesis.

### Data extraction

Each article/document was given a meta-data code relating to its database source. All the titles were gathered in Microsoft Excel (Microsoft 365 MSO Version 2207) (RRID:SCR_016137) and assessed according to the critical appraisal strategy. Meta-data extracted included the title, year of publication, country of first author institution, country/countries where study was conducted, and whether these were in ‘Global South’ or ‘Global North’. Data was also gathered to assess whether the article described or assessed: ‘volunteer experience’, ‘volunteer motivation’, ‘positive impacts on or benefits to volunteers’, ‘impacts on the community’ and’barriers to or negative impacts on volunteers’. Not all articles would cover all these topics and in the absence of content, the Excel sheet cell was left blank. Raw data were stored in Figshare. ^27^

### Data synthesis and presentation

The type of synthesis conducted as part of the systematic review will be a combination of narrative and mixed method syntheses. Descriptive statistics, figures and tables will be used to synthesise the evidence base where appropriate, namely the number, quality, year of publication and geographic spread of articles and other documents collated. This will include assessment of the effect of region and country of the study location as well as the country where the institution or organisation of the first author is based.

Findings will be amalgamated under different themes including:

1) Motivations for volunteering
2) Barriers to participating
3) Perceived impacts from volunteering activities

A narrative, qualitative synthesis form will be used to describe and group different motivations, barriers and impacts identified in the various studies. Not all sources will have covered each of these themes so sample sizes for each will be listed and implications discussed.

The second part of the study will describe the variety of methods used to gather and assess the above data. Literature reviews, systematic or otherwise, identified in the search phase, will be collated and their findings will be presented using a narrative synthesis. The risk of publication bias will be discussed alongside the strategy to identify potential knowledge gaps and the unrepresented subtopics that may warrant further primary research. ^24^

## Data availability

### Underlying data

Figshare: Underlying data for ‘Assessing the impacts of conservation volunteering on participant well-being: a simplified systematic review protocol’, https://doi.org/10.6084/m9.figshare.19525615.v1. ^27^

Data are available under the terms of the Creative Commons Zero “No rights reserved” data waiver (CC0 1.0 Public domain dedication).

### Reporting guidelines

PRISMA-P checklist for ‘Assessing the impacts of conservation volunteering on participant well-being: a simplified systematic review protocol’, https://doi.org/10.6084/m9.figshare.21030238. ^26^

Data are available under the terms of the Creative Commons Attribution 4.0 International license (CC-BY 4.0).
