# Supplementary material for: Assessing the impacts of conservation volunteering on participant wellbeing: a systematic review protocol
Source: F1000Res. 2023 Mar 6;11:1136. Originally published 2022 Oct 4. [Version 2] doi: 10.12688/f1000research.113630.2 (PMC10020737; doi:10.12688/f1000research.113630.2)
Supplement: Peer review history

# Reviewer report: Reviewer response for version 2

**DOI:** 10.5256/f1000research.143949.r165575

**Published:** 2023-03-16

**Authors:** Philip A. Martin

**Affiliations:** 1 Basque Centre for Climate Change, Leioa, Spain

Having looked at the responses to my review I am satisfied that the authors have addressed my concerns. Based on these revisions I am happy for the work to be indexed.

Is the study design appropriate for the research question?

Partly

Is the rationale for, and objectives of, the study clearly described?

Yes

Are sufficient details of the methods provided to allow replication by others?

Partly

Are the datasets clearly presented in a useable and accessible format?

Yes

Reviewer Expertise:

NA

I confirm that I have read this submission and believe that I have an appropriate level of expertise to confirm that it is of an acceptable scientific standard.

# Reviewer report: Reviewer response for version 2

**DOI:** 10.5256/f1000research.143949.r165576

**Published:** 2023-03-10

**Authors:** Margaret Nohilly, Fionnula Tynan

**Affiliations:** 1 Department of Learning, Society and Religious Education, Mary Immaculate College, Limerick, Ireland; 2 Mary Immaculate College, Limerick, Ireland

Having reviewed the revised document here and in word form, the author has taken time to incorporate many of the recommendations which strengthen this paper overall

Is the study design appropriate for the research question?

Yes

Is the rationale for, and objectives of, the study clearly described?

Yes

Are sufficient details of the methods provided to allow replication by others?

Yes

Are the datasets clearly presented in a useable and accessible format?

Yes

Reviewer Expertise:

Wellbeing, Child Protection

We confirm that we have read this submission and believe that we have an appropriate level of expertise to confirm that it is of an acceptable scientific standard.

# Reviewer report: Reviewer response for version 1

**DOI:** 10.5256/f1000research.125364.r157464

**Published:** 2023-01-03

**Authors:** Philip A. Martin

**Affiliations:** 1 Basque Centre for Climate Change, Leioa, Spain

This study aims to assess the impacts of conservation volunteering on the wellbeing of the participants using systematic review to synthesise the findings of previous studies. This is an interesting question, particularly, as the author states, in light of current attempts to scale up ecosystem restoration.

 I am a specialist in ecological synthesis and have approached my review from this perspective, using the Collaboration for Environmental Evidence checklist for editors and reviewers and a guide ( https://environmentalevidence.org/wp-content/uploads/2021/02/CEESAT-Checklist-for-editors.pdf). Based on this, I conclude that this synthesis is likely to be significantly better than the vast majority of synthesis papers published in ecology and conservation. For example it was very good to see (i) a well-defined series of questions; (ii) a flowchart conceptualising the potential impacts of volunteering; (iii) use of a PRISMA diagram; (iv) consistency checks for inclusion criteria; (v) critical appraisal of studies. However, I still think that there are a number of issues that need to be resolved.

 Major issues

- I am a bit confused about what this protocol is for. Normally, a protocol is written before a study is carried out and pre-registered in order to avoid questionable research practices. However, in the case of this study it seems like much of the study has already been carried out. Is that the case? If so, what is the purpose of this document.
- It’s fantastic that a good number of platforms were used to search for relevant literature, however it’s not clear to me what the search strings used for platforms other that Web of Science were. In order to make this work as repeatable as possible it would be helpful if detail on this was provided.
- For the screening, how was it decided that titles were irrelevant? I’m also unclear as to whether the inclusion criteria were applied at the abstract stage or the full text stage of screening or both. It would be great if you could clarify this.
- The critical appraisal strategy is missing detail. It would be helpful for me if all the of criteria for critical appraisal were presented in a table so it is clearer what the impact of different scores for each category are for the overall scores for internal and external validity.

Minor issues

- In the question component section some mention of PICO elements would be helpful.
- I can’t tell what tool was used for the deduplication of references that were found in searches. Can you please provide some detail on this?
- Why were papers restricted to those published between 2000 and 2020? In addition, although this is mentioned in the abstract, it is not mentioned anywhere in the main text of the article. This information should be included for the sake of clarity.

Typos

- In the section on searches ‘Scobus’ should be ‘Scopus’

Is the study design appropriate for the research question?

Partly

Is the rationale for, and objectives of, the study clearly described?

Yes

Are sufficient details of the methods provided to allow replication by others?

Partly

Are the datasets clearly presented in a useable and accessible format?

Yes

Reviewer Expertise:

Ecological evidence synthesis

I confirm that I have read this submission and believe that I have an appropriate level of expertise to confirm that it is of an acceptable scientific standard, however I have significant reservations, as outlined above.

# Response

**Published:** 2023-01-25

**Authors:** Hanna Nuuttila

**Affiliations:** Swansea University, UK

Major issues

- I am a bit confused about what this protocol is for. Normally, a protocol is written before a study is carried out and pre-registered in order to avoid questionable research practices. However, in the case of this study it seems like much of the study has already been carried out. Is that the case? If so, what is the purpose of this document.

You are correct. The study protocol was started just after the covid pandemic broke out and left me, the main and only researcher, in a difficult position without much academic support from my institution. The protocol was written but not published for various reasons whilst time and funding to conclude the whole work was running out (this was a short fellowship). I was unable to conclude the intended research as face to face opportunities shrunk and the only thing I could do was to continue with the systematic review – following the guidelines I had written (but not published). As you say – the protocol’s purpose now is just to support work that has already been started – however the findings have not been analysed or written up and all comments and suggestions for the methods will be useful – even if modifications into search methods etc cannot be made. These can, and will be discussed in the research paper – and I must say I have learned a great deal about systematic reviews, protocols and publication.

  

- It’s fantastic that a good number of platforms were used to search for relevant literature, however it’s not clear to me what the search strings used for platforms other that Web of Science were. In order to make this work as repeatable as possible it would be helpful if detail on this was provided.

I have modified and amended this and hopefully this is now clearer:

 The search strings used are listed below:

*“conservation” OR “community conservation” OR “community based conservation” OR “community-based conservation” OR “volunteer conservation” OR “participatory conservation” OR “citizen science” AND “volunteer” OR “volunteering” OR “wellbeing” OR “well-being”*

 In addition, limited searches were conducted for “impacts of conservation”, “impacts of conservation volunteering”, “impacts of community-based conservation”, “co-benefits of conservation”, “co-benefits of conservation volunteering”, “conservation volunteering AND “happiness”, “conservation volunteering AND “health” to check how well the general search string were capturing the intended literature.

  

- For the screening, how was it decided that titles were irrelevant? I’m also unclear as to whether the inclusion criteria were applied at the abstract stage or the full text stage of screening or both. It would be great if you could clarify this.

Title screening consisted of checking the title against the following criteria and whether it alluded that the paper would discuss any of the following: conservation/restoration/rewilding project/natural resource management / volunteer participants/community conservation/ community participation/citizen science / impacts or wellbeing linked to conservation, environment and volunteering in any form / challenges, barriers, motivations, attitudes, or incentives to volunteering or community based conservation or participatory experience.

 If it wasn’t not clear whether or not the article might discuss the above, it was kept for further screening.

 In addition, I included for further reading, titles that alluded to the above topics which could be discussed in the text, specifically when these were titled as reviews or analyses.

 I will add the above to the methodology for clarification

  

- The critical appraisal strategy is missing detail. It would be helpful for me if all the of criteria for critical appraisal were presented in a table so it is clearer what the impact of different scores for each category are for the overall scores for internal and external validity.

Research quality matrix. Table not visible in this format and will be added to main text.

**EXTERNAL VALIDITY**

 (sample size)

**INTERNAL VALIDITY**

 (extent, repeatability and clarity of methodology)

 < 20 (low)

 > 20 (high)

 Not described (no)

 LOW

 NO

 HIGH

 NO

 Limited description (lim)

 LOW

 LIM

 HIGH

 LIM

 Adequate description (ade)

 LOW

 ADE

 HIGH

 ADE

 Matrix results for data used in the analysis.

 EXCLUDE

 REVIEW

 INCLUDE

 Minor issues

- In the question component section some mention of PICO elements would be helpful.

Below you can see the text from the methods which does identify the PICO elements, I have added codes in brackets for clarity – and repharsed the Main research question with coded elements as follows:

**What are the impacts (O) (positive or negative) of voluntary participation (I)  in conservation and restoration activities to the participating volunteers (P)?**

 The **subject/population (P), **or the unit of study was any identified habitat or species conservation or restoration project with a definite aspect of community and/or volunteer participation described in the literature.

 The **intervention (I)** was the volunteer participation, and the **outcome (O)** was the effects on participants’ or perceived mental, physical, social and economic wellbeing, both at individual as well as community level.

 The review’s focus was specifically the described (subjective) perceptions of individuals who have participated in volunteering activities. As such, it was not possible to find a suitable **comparator**, such as review of volunteering impacts from non-conservation projects, that would have provided adequate, reliable data for comparison.

 I can’t tell what tool was used for the duplication of references that were found in searches. Can you please provide some detail on this?The ‘duplicate’ search in Excel was used, as was organising in alphabetical order and visually checking for duplicates.  Will add this text to the methods for clarification

  

- Why were papers restricted to those published between 2000 and 2020? In addition, although this is mentioned in the abstract, it is not mentioned anywhere in the main text of the article. This information should be included for the sake of clarity.

Thanks, will add this information. 20 years’ of datasets was deemed something I could tackle by myself, also In pilot searches the mention of wellbeing was simply not coming up in earlier papers, and knowing

 Typos

- In the section on searches ‘Scobus’ should be ‘Scopus’

This will be corrected

# Reviewer report: Reviewer response for version 1

**DOI:** 10.5256/f1000research.125364.r152400

**Published:** 2022-10-25

**Authors:** Margaret Nohilly, Fionnula Tynan

**Affiliations:** 1 Department of Learning, Society and Religious Education, Mary Immaculate College, Limerick, Ireland; 2 Mary Immaculate College, Limerick, Ireland

This is a very interesting article with a focus on volunteers, which as the article outlines is not often the focus of a research project. The article focuses in particular on the impact of conservation activities on participating volunteers. A clear rational is provided for the study and the objectives of the research are clearly outlined. 

 I recommend that a stronger focus is placed on the concept of wellbeing in this work, particularly in line with secondary research question: If 'Wellbeing' is discussed, described, or assessed, how has it been defined? What aspects of wellbeing have been included? The concept of wellbeing needs to be explored in detail and made accessible to the reader. The conceptual framework outlines a conceptual framework for mental wellbeing. Does this not indicate that mental wellbeing is wellbeing? Mental wellbeing is a component of one of many subsets of wellbeing no more than physical wellbeing, spiritual wellbeing, emotional wellbeing are subsets of wellbeing. I recommend that a more holistic view of wellbeing is explored and presented as a conceptual framework for wellbeing in this article.

 The protocol for undertaking the work must be commended as it is well detailed and robust.

Is the study design appropriate for the research question?

Yes

Is the rationale for, and objectives of, the study clearly described?

Yes

Are sufficient details of the methods provided to allow replication by others?

Yes

Are the datasets clearly presented in a useable and accessible format?

Yes

Reviewer Expertise:

Wellbeing, Child Protection

We confirm that we have read this submission and believe that we have an appropriate level of expertise to confirm that it is of an acceptable scientific standard, however we have significant reservations, as outlined above.

# Response

**Published:** 2023-01-25

**Authors:** Hanna Nuuttila

**Affiliations:** Swansea University, UK

I completely agree that I had not defined the concept adequately. I have conducted much more exploration of the concept. The study proposes to understand the ways in which ‘wellbeing’ has been conceptualised and measured in the field of conservation and following is something I wrote for another paper. I will utilise this to form a more complex, multifaceted definition of the concept for the methods paper.

 ‘Human wellbeing’ can be defined in a variety of ways.

 As our understanding of wellbeing has evolved in psychology, sociology and economics so has our way of defining and assessing it in conservation practice and policy. Understanding human wellbeing is now a critical component in sustainably managing the environment. Biedenweg et al. identify six human wellbeing indicators (psychological, physical, social, cultural, economic, governance) which are affected by the environment and as such are useful in guiding policy and management (Biedenweg, Stiles, and Wellman 2016).

 It is generally accepted that human wellbeing is composed of both objective and subjective dimensions (Summers et al. 2012). The objective components are related to material and social attributes and are easily measurable such as our jobs and incomes, homes, places we live in, our environment, available services, infrastructure so on. Equally essential part is the subjective, perceived wellbeing that can only be described by the individual. This relates to job or health satisfaction, our feelings of belonging, empowerment, available choices as well as our subjective measure of mental wellbeing, our relationships and trust in society and civic institutions around us.

 Understanding of wellbeing has evolved from ‘basic human needs’ to encompass ecosystem services, environmental needs and subjective happiness (Summers et al. 2012). We now define ‘wellbeing’ through how people are feeling and functioning in their everyday lives.

 Crucially we now acknowledge that wellbeing is a social and cultural construct and aspects of wellbeing can differ drastically geographically, even if some components are universally accepted (Beauchamp et al. 2018; de Lange, Woodhouse, and Milner-Gulland 2016; McKinnon et al. 2016).  

 The Office of National Statistics (ONS) in the UK compiles data on ten domains of national wellbeing[1]; personal well-being; our relationships; health; what we do; where we live; personal finance; economy; education and skills; governance and environment. Within these 10 domains there are 44 indicators of national well-being. The indicators include both objective measures (for example, unemployment rate) and subjective measures (such as job satisfaction) to provide a comprehensive picture of the nation's well-being and ‘societal progress’. In contrast, a wellbeing definition used in a conservation project in a rural Tanzania measures wealth in tropical livestock units, assesses relational and social wellbeing through ability to attend community meetings and places a far more weight and importance in the continuity of traditional ways of life, spiritual practice or female autonomy (Beauchamp et al. 2018). At its most simplistic, human wellbeing in conservation has been defined through three conditions: meeting needs, pursuing goals and experiencing a satisfactory quality of life (Milner-Gulland et al. 2014).

 Here, we define wellbeing as a multidimensional concept, incorporating all aspects of our lives including social and cultural, environmental and physical, economic and material, and psychological including emotional and spiritual wellbeing. Wellbeing has both objective and subjective aspects. Objective wellbeing is something we theoretically assume can be measured, whereas subjective wellbeing is an externally experienced concept that only the individual can report on. Happiness and wellbeing are sometimes used interchangeably, but constant happiness is not necessarily a prerequisite to one’s general wellbeing. Here the feeling of happiness is considered as one of the elements of wellbeing – but as a momentary and changeable emotion. It is possible to feel sad and have wellbeing simultaneously, just as it is possible to be of ill health or in inadequate economic situation whilst feel happy or content. This complexity makes measuring wellbeing in its entirety difficult, and is probably the reason why many wellbeing studies aimed at assessing wellbeing focus on limited aspects of wellbeing.

 [1] Office for National Statistics (ONS), released 11 Nov 2022, ONS website, Statistical bulletin: Quality of life in the UK: November 2022
